# Derivation of Mouse Dome‐Shaped Trophoblast Stem Cells With Low Heterogeneity From Blastocysts

**DOI:** 10.1111/cpr.70280

**Published:** 2026-09-18

**Authors:** Minli Liu, Shaofang Ren, Xinyu Xia, Guanlin Lu, Haojie Yang, Yi Zheng, Jiexiang Zhao, Xiao‐Yang Zhao, Mei Wang

**Affiliations:** ^1^ State Key Laboratory of Multi‐Organ Injury Prevention and Treatment, Department of Developmental Biology, School of Basic Medical Sciences Southern Medical University Guangzhou Guangdong China; ^2^ Key Laboratory of Tropical Translational Medicine of Ministry of Education, School of Basic Medical Sciences, Hainan Academy of Medical Sciences Hainan Medical University Haikou Hainan China; ^3^ School of Laboratory Medicine and Biotechnology Southern Medical University Guangzhou Guangdong China; ^4^ Key Laboratory of Mental Health of the Ministry of Education Guangzhou Guangdong China; ^5^ Guangdong Provincial Key Laboratory of Construction and Detection in Tissue Engineering Southern Medical University Guangzhou Guangdong China; ^6^ Guangdong‐Hong Kong Joint Laboratory for Psychiatric Disorders Guangzhou Guangdong China; ^7^ Department of Gynecology, Zhujiang Hospital Southern Medical University Guangzhou Guangdong China; ^8^ National Clinical Research Center for Kidney Disease Guangzhou Guangdong China

**Keywords:** differentiation, heterogeneity, mouse, placenta, trophoblast, trophoblast stem cells

## Abstract

Trophoblast stem cells (TSCs) have emerged as a valuable model for investigating placentation and related disorders. Despite their ability to be maintained over multiple passages without losing stemness, conventional TSCs are characterised by notorious heterogeneity and a spontaneous differentiation tendency. In this study, we established a type of mouse TSCs with dome‐shaped colonies (dTSCs). The dTSCs expressed canonical markers of conventional TSCs and exhibited relatively low levels of heterogeneity and spontaneous differentiation when cultured in conventional trophoblast stem cell medium. Additionally, dTSCs retained robust potential to differentiate into all trophoblast lineages in vitro, as well as the capacity to contribute to placental development in vivo. Furthermore, the functions of *Foxo4* and *Slc16a3* (MCT4) could be effectively explored in trophoblast development by using our established dTSCs. Collectively, our findings establish a type of high‐quality TSCs and define dTSCs as a promising and refined model for investigating trophoblast development.

## Graphical abstract

Here, Liu et al. report the generation of mouse dome‐shaped trophoblast stem cells (dTSCs) with low heterogeneity and autonomous differentiation potential. dTSCs robustly generate all trophoblast lineages in vitro and contribute to the placenta in vivo, providing a refined model for studying trophoblast development and related disorders.

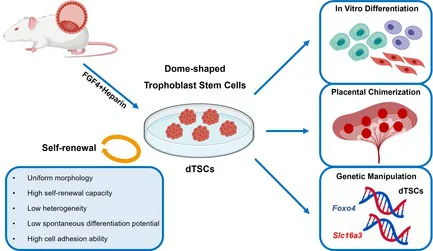

## 1 Introduction

The placenta is a transient but vital organ that supports foetal development by producing hormones that act on maternal tissues, facilitating the exchange of gases and nutrients, and providing immunological protection at the maternal‐foetal interface [1, 2, 3]. The extra‐embryonic trophoblast lineage gives rise to the major constituents of the placenta, and its differentiation defects often result in abnormal placental development and dysfunction, which frequently lead to a variety of human pregnancy‐associated diseases, for example, recurrent spontaneous abortion (RSA), preeclampsia (PE), and foetal growth restriction (FGR) [4, 5, 6, 7].

Trophoblast stem cells (TSCs) are considered the in vitro counterparts of placental precursor cells. TSCs are derived from the polar trophectoderm (pTE) of the blastocyst or the extraembryonic ectoderm (ExE) [8, 9, 10, 11, 12, 13]. When cultured under appropriate conditions, mouse TSCs can indefinitely self‐renew, retain multipotency to differentiate into all specialised trophoblast cell types, and contribute to chimeric placentas after injection into early embryos [8, 11, 14]. TSCs endow us with unprecedented possibilities to investigate the molecular, biochemical, genetic, and environmental aspects that direct trophoblast proliferation and differentiation in vitro [15, 16, 17].

Currently, robust culture systems for mouse TSCs have been established utilising either serum‐based or chemically defined media [8, 10]. However, it is evident from the morphology and gene expression level within the colonies that, even under optimal conditions, mouse TSCs exhibit a significant degree of heterogeneity [18, 19, 20, 21, 22, 23]. A previous study demonstrated that colonies of TSCs could be classified into four major types: Type 1 is compact and dome‐shaped, Type 4 is flattened but with a large multilayered cell cluster, and Types 2 and 3 are their intermediates. Time‐lapse analysis indicated that Type 1 colonies predominantly emerge following passaging [20]. In addition, it is very difficult to completely avoid spontaneous differentiation of TSCs even in stem cell conditions. The transcriptomic profiles indicate that a small subset of TSCs has already initiated differentiation and changed their cell cycle dynamics [23, 24]. Therefore, it is essential to establish high‐quality TSCs with low heterogeneity.

Here, we established dome‐shaped trophoblast stem cell lines (dTSCs), which were distinct from conventional flattened TSCs (cTSCs). These dTSCs exhibited relatively low heterogeneity and minimal spontaneous differentiation in conventional medium. Moreover, the ability of dTSCs to differentiate into various trophoblast lineages in vitro and to contribute to the placenta in chimeras remained intact. These findings highlight the significance of dTSCs in enhancing the quality of trophoblast stem cell lines and provide an ideal model for investigating the regulatory networks of trophectoderm as well as placental development.

## 2 Materials and Methods

### 2.1 Ethics Approval and Consent to Participate

Animal procedures were performed according to the ethical guidelines of the South Medical University Animal Ethics Committee (No. L2021162).

### 2.2 Mice

ICR (CD‐1) mice were used in the study and maintained under specific pathogen‐free (SPF) animal facility at Southern Medical University. They were subjected to a 12‐h light/dark cycle, with temperature controlled between 20°C and 25°C and humidity maintained at 40%–70%. Mice were free to access water and food.

### 2.3 Derivation and Cultivation of TSCs

When a copulation plug was present on the following morning (before 9 AM), that time point was recorded as embryonic Day 0.5 (E0.5). E3.5 blastocysts were isolated from pregnant ICR females. Placing them in mitotically inactivated CF1 mouse embryonic fibroblast (MEF)‐coated plates with TSC medium [8, 25]. TSC medium: advanced RPMI 1640 (11875‐093, Thermo Fisher Scientific) supplemented with 20% (v/v) foetal bovine serum (FBS) (SE100‐011, VisTech), 25 ng/mL recombinant human FGF4 (235‐F4‐025, R&D), and 1 μg/mL heparin (07980, Stem Cell). The outgrowths were monitored daily and passaged on Days 6–10 depending on cell growth. Established mouse TSC lines were cultured on MEF plates in TSC medium. Cells were cultured at 37°C under 5% CO_2_, with the culture medium renewed every 48 h. The conventional TSC cell line (CD‐1 strain) was provided by Pro. Xuefei Gao (Southern Medical University).

Colony morphological analysis of cTSCs and dTSCs was carried out based on the previous study [20]. A total of six independent experiments were conducted, and approximately 20 colonies were randomly selected for analysis in each experiment.

### 2.4 Culture of Embryonic Stem Cells (ESCs) or Extended Pluripotent Stem Cells (EPSCs)

GFP‐ESCs were derived from the E3.5 blastocysts of C57BL/6‐Tg (CAG‐GFP) mouse, which is from our laboratory. GFP‐ESCs were cultured in N2B27 basal medium [26, 27] supplemented with 1000 IU LIF (130‐099‐895, Miltenyi Biotec), 1 μM PD0325901 (4423, Tocris), and 3 μM CHIR99021 (4192, Tocris). ESCs were cultured at 37°C and 5% CO_2_ on MEF‐coated tissue culture plates and passaged when they reached 80% confluency [28, 29].

To generate GFP‐EPSCs, we cultured GFP‐ESCs in EPSC medium on feeder layers for over 20 passages, and termed them EPSCs. EPSC medium: N2B27 basal medium supplemented with 10 ng/mL recombinant human LIF (300‐05, Peprotech), 3 μM CHIR99021, 2 μM (S)‐(+)‐dimethindenemaleate (1425, Tocris), and 2 μM Minocycline hydrochloride (sc‐203339, Santa Cruz Biotechnology) [29, 30].

### 2.5 Cell Cycle Analysis

TSCs grown in TSC medium were dissociated by incubation (5 min) in 0.25% trypsin and fixed with 70% ice‐cold ethanol. Cells were stained in propidium iodide (PI) staining solution (6.7–33.4 μg/mL PI in PBS) for 30 min at room temperature. Flow cytometric analysis was performed on a FACSCanto (BD Biosciences) and analysed using FlowJo Software.

### 2.6 Karyotype Analysis

Karyotype analysis was performed as described previously [31]. When the cells reached 70%–90% confluence, 0.8 μg of colchicine was added, and cells were incubated for 1 h. The cell suspension was collected and centrifuged to remove the supernatant. A total of 10 mL of pre‐warmed 0.56% KCl hypotonic solution was slowly added to the centrifuge tube, followed by incubation at 37°C for 30 min. One millilitre of pre‐cooled, freshly prepared methanol/glacial acetic acid fixative (methanol: glacial acetic acid = 3:1) was added, the mixture was centrifuged at 1200 rpm for 5 min, and the supernatant was discarded. Subsequently, 10 mL of pre‐cooled fixative was added again, and cells were fixed on ice for 15 min, centrifuged at 1200 rpm for 5 min, and the supernatant was removed. This step was repeated twice. Cells were resuspended in 100 μL of pre‐cooled fixative and dropped from a height onto pre‐chilled glass slides stored at −20°C. Slides were then stained with Giemsa solution for 20 min. Images were obtained with a microscope (Zeiss, Axio Imager.A2).

### 2.7 Immunostaining

Immunostaining was performed as described previously [32]. Cells and placental paraffin sections were blocked with 5% bovine serum albumin (BSA) for 1 h and then incubated with primary antibodies overnight at 4°C. Subsequently, the samples were washed three times with PBS, followed by incubation with secondary antibodies for 1 h at room temperature. The nuclei were counterstained with 10 μg/mL Hoechst 33342 for 15 min at room temperature, followed by washing with PBS. Images were captured with a ZEISS LSM880 confocal microscope. The antibody details were listed in Table S1.

### 2.8 Haematoxylin and Eosin (H&E) Staining

E12.5 mouse placentas were fixed in 4% paraformaldehyde at 4°C for 24 h, paraffin‐embedded, and sectioned at 5 μm. After xylene deparaffinisation and ethanol gradient rehydration, sections were sequentially stained with haematoxylin (5 min) and eosin (10 min). Micrographs were acquired on a Carl Zeiss microscope.

### 2.9 Quantitative Real‐Time PCR (qPCR)

Total RNA was extracted using the TRIzol reagent. Reverse transcription was performed by Hiscript III Reverse Transcriptase. qPCR was conducted utilising the qPCR SYBR Green Master Mix and Bio‐Rad CFX96 Real‐Time System (Bio‐Rad CFX Manager 3.1 software). Relative mRNA expression was quantified employing the Delta–Delta CT method and normalised to *Gapdh*. Primers in qPCR assays were listed in Table S2.

### 2.10 Western Blotting Analysis

Western blotting analysis was performed as previously described [33]. TSC cells were prepared using cold RIPA for 30 min. Protein lysates were separated by SDS‐PAGE and transferred to nitrocellulose membranes. Membranes were blocked with 5% skim milk in TBST (20 mM Tris, 150 mM NaCl, pH 7.6 with 0.05% Tween‐20) and incubated with the primary antibody overnight. The next day, membranes were washed with TBST buffer for 5 min and incubated with HRP‐conjugated secondary antibodies at room temperature for 1 h. Signals were visualised by an enhanced chemiluminescence detection system and analysed by ImageJ. The antibody details were listed in Table S1.

### 2.11 tdTomato‐Labelled Cell Line Generation

tdTomato‐labelled mouse TSCs were generated by targeted integration using the homology‐mediated end joining based strategy [34]. Donor vectors were constructed as depicted in the laboratory literature to introduce the CAG‐P2A‐tdTomato cassette into the *Rosa26* locus [35]. Unique gRNA sequence targeting the *Rosa26* locus was the same as previously described [34]: 5′‐ACTCCAGTCTTTCTAGAAGA‐3′. And Oligos encoding gRNA were inserted into pX458 vector [36]. Then 7–10 μg donor vector and 7 μg pX458 vector containing sgRNA targeting the *Rosa26* locus were introduced into 1 × 10^6^ TSCs via electroporation (MPK5000, Invitrogen), and followed by selection with puromycin (0.3 μg/mL, S7417, Selleck).

### 2.12 Flow Cytometry Doublet Assay

Differentially labelled cells (mouse EPSCs expressing GFP and cTSCs/dTSCs labelled with tdTomato) were combined at a ratio of 1:1 for a total of 200,000 cells per well. This cell mixture was plated in a 24‐well low‐adhesion dish (Corning) and agitated at 80 rpm on an orbital shaker at 37°C for 1 h. The cells were filtered through a 70 μm cell strainer to break up large clusters and analysed using a MoFlo XDP flow cytometer [37]. We used an expanded live‐cell gate to capture doublets, and only the resulting doublets were analysed for GFP and tdTomato fluorescence using FlowJo software. In these doublet assays, cells were cultured in FTW medium [38].

### 2.13 Mouse Stem Cells Co‐Culture Assay

GFP‐ESCs and tdTomato‐cTSCs/dTSCs were seeded onto MEF‐coated plates either cultured separately or mixed at different ratios for co‐cultures. A starting number of GFP‐ESCs (1.5 × 10^4^ cells) and tdTomato‐TSCs (3 × 10^4^ cells) were decided on for most of the cell–cell co‐culture assays. During co‐culture experiments, cells were cultured in FTW medium on MEFs for 4 days and the following analyses were performed [38, 39].

For all of the co‐culture experiments, total GFP‐ESCs or tdTomato‐TSCs numbers were quantified by the following method [38]. Cells were dissociated into single cells using 0.25% trypsin at 37°C for 3–5 min, and the total number of live cells was counted. Next, the relative percentage of GFP or tdTomato fluorescence was determined using an LSR II Flow Cytometer (BD Biosciences). The total ESC or TSC cell number (*tN*) for each group under both the co‐cultured and separate culture conditions was determined by multiplying total cell volume (*V*) by the cell concentration (*CC*) and the percentage of GFP^+^ or tdTomato^+^ cells (P).

tN=V×CC×P.

### 2.14 shRNA Transfection

*Foxo4* and *Slc16a3* were knocked down in dTSCs by lentiviral delivery of shRNAs. Plasmids encoding pHIV‐CopGFP‐F2A‐puro or pHIV‐CopGFP‐F2A‐puro‐shRNAs were obtained from Ribo Biotechnology. Lentiviral packaging vectors from Addgene (pMDLg/pRRE Plasmid #12251, pRSV‐Rev Plasmid #12253, and pMD2.G Plasmid #12259) were used to produce the viral particles in HEK293T cells as described earlier. Culture supernatants containing lentiviral particles were harvested every 24 h for 2 days, centrifuged to remove cell debris, filtered, and applied to TSCs in culture. Transduced cells were selected for puromycin resistance. Functionally active shRNA sequences were shown in Table S3.

### 2.15 RNA Isolation and Library Preparation

Total RNA isolation and Illumina transcriptome sequencing library preparation were performed according to the manufacturer's instructions with the NEB Next Ultra II Directional RNA Library Prep Kit (E7760L, NEB). The constructed NGS libraries were sequenced with the Illumina Hiseq XTEN platform at Novogene and the sequencing strategy was paired‐end 150 bp (PE150).

### 2.16 Quality Control of RNA Sequencing (RNA‐Seq) Data

The original fluorescence image files obtained from the Illumina platform were transformed to short reads via base calling, and these short reads were recorded in FASTQ format, which contains sequence information and corresponding sequencing quality information. Fastp (version 0.23.1) was subsequently used to perform basic statistics on the quality of the raw reads [40]: (1) Discard paired reads if one read contains adapter contamination; (2) Discard paired reads if more than 10% of bases are uncertain in one read; and (3) Discard paired reads if the proportion of low‐quality (Phred quality < 5) bases is greater than 50% in one read.

### 2.17 Analysis of Differentially Expressed Genes (DEGs)

Clean reads obtained after quality control were aligned to the reference genome using HISAT2 (version 2.2.1) [41]. Gene‐level counts were quantified with featureCounts (version 2.0.1). The raw count matrix was imported into DESeq2 (version 1.34.0), and size factors were estimated using the package's internal median‐of‐ratios method to normalise for library size differences [42]. Normalised counts were used to calculate Spearman correlation coefficients, and hierarchical clustering was performed using the hclust function in R to assess sample similarity. DEG analysis between groups was conducted using DESeq2's Wald test, with Benjamini‐Hochberg correction applied to control the false discovery rate [43]. Genes with |log_2_(fold change)| > 1 and *p* < 0.05 were defined as DEGs. Gene Ontology (GO) enrichment analysis of DEGs was performed using Metascape with default parameters. Kyoto Encyclopedia of Genes and Genomes (KEGG) pathway enrichment analysis was conducted using DAVID Bioinformatics Resources. Enriched GO terms and KEGG pathways were ranked according to statistical significance after multiple‐testing correction, and *p* < 0.05 was considered statistically significant. All analyses were carried out in R (version 4.1.2).

### 2.18 Embryonic Injection and Transplantation

tdTomato or GFP‐positive TSCs were dissociated and resuspended in M2 (M7167‐100, Sigma) and TSC basal mix medium at 4°C. Approximately 10 positive TSCs were microinjected into each 8‐cell stage embryo to produce chimeric blastocysts. After 16–20 h, the manipulated blastocysts were transferred into the oviducts of E0.5 pseudo‐pregnant recipients. The total number of unilateral uterine transfer blastocysts in each ICR mouse was no more than 10. Implantation was examined by gentle exfoliation of the conceptus in PBS 12.5 days after embryo transfers.

### 2.19 Quantification and Statistical Analysis

All experiments were performed with at least three independent biological replicates. Statistical analysis was performed using GraphPad Prism (version 9.5.0). The significance of differences between two groups was analysed by unpaired two‐tailed Student's *t*‐test. The significance of differences among three or more groups was analysed by one‐way ANOVA. Quantitative data are presented as mean ± SD. A *p*‐value less than 0.05 was considered to indicate a significant difference.

## 3 Results

### 3.1 Derivation of Mouse Dome‐Shaped TSC Lines From E3.5 Blastocysts

**FIGURE 1:**
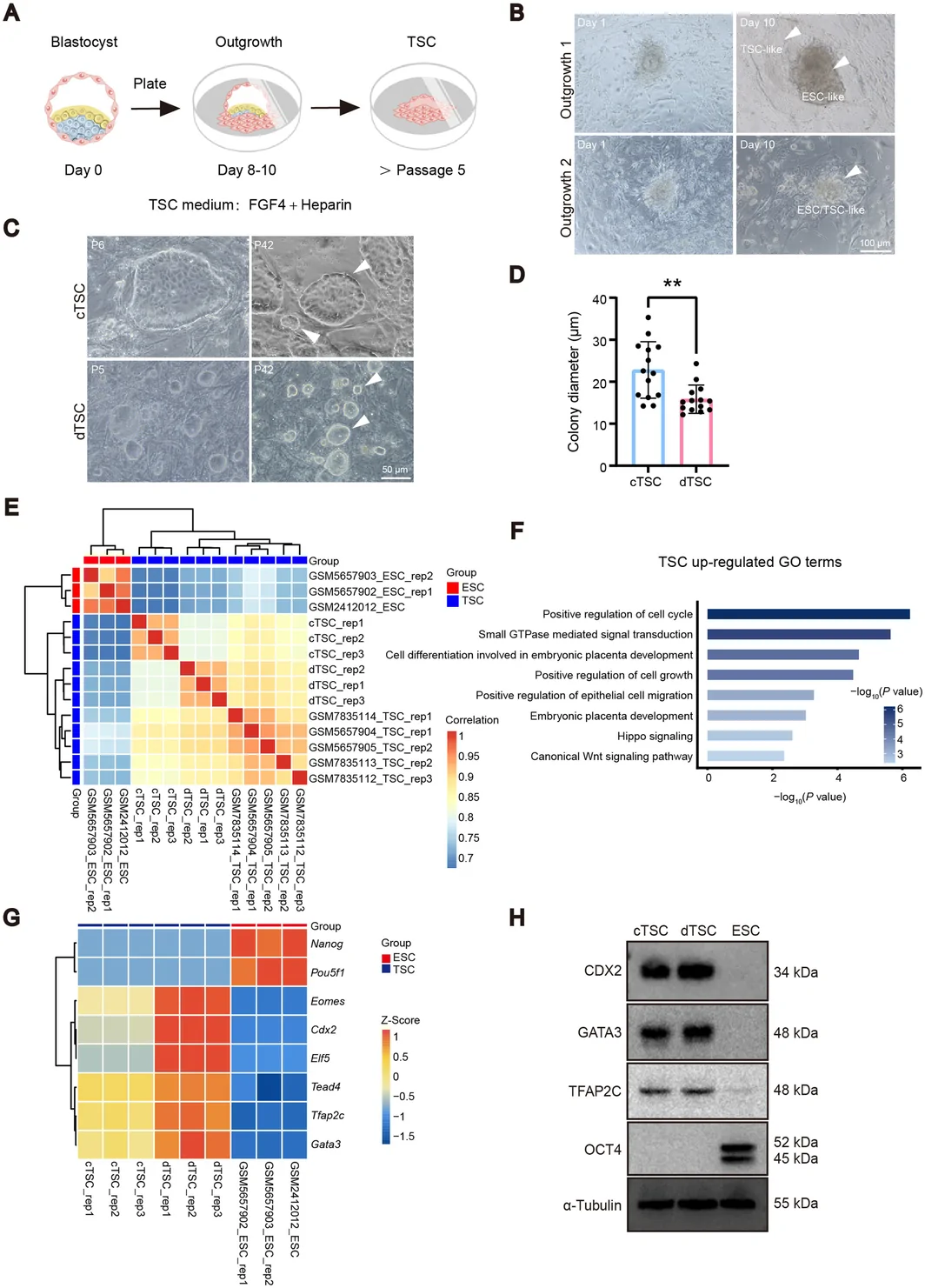
Derivation of mouse dome‐shaped TSC lines from E3.5 blastocysts. (A) Illustration of the derivation of TSC lines from mouse E3.5 blastocysts. (B) Morphology of outgrowths derived from blastocysts at Day 1 and day 10.TSC‐like, trophoblast stem cell‐like; ESC‐like, embryonic stem cell‐like. Scale bar, 100 μm. (C) Bright field images of dTSCs at passage 5 (P5) and passage 42 (P42). cTSCs are used as control. Scale bar, 50 μm. (D) Colony diameter analysis of cTSCs and dTSCs. Data are presented as mean ± SD (*n* = 14 per group); unpaired two‐tailed Student's *t*‐test; **p < 0.01. (E) Hierarchical clustering heatmap showing the Spearman correlations across cTSCs, dTSCs, and ESCs (*p* < 0.05, 2‐fold change). A gradient of blue to red indicates low to high correlations. (F) GO enrichment analysis of common up‐regulated DEGs in dTSCs and cTSCs relative to ESCs. The colour gradient indicates the significance level (*p*‐value). The criteria for DEGs are defined as log_2_(fold change) > 1 and *p* < 0.05. (G) Heatmap analysis of gene expression profiles in cTSCs, dTSCs, and ESCs. The colour key from blue to red indicates low to high gene expression levels, respectively. Data are *Z*‐score transformed prior to clustering. (H) Western blotting analysis of CDX2, GATA3, TFAP2C, and OCT4 in cTSCs, dTSCs, and ESCs, with α‐Tubulin as the internal control.

In our study, 12 TSC lines were established from 49 E3.5 ICR blastocysts using conventional TSC medium containing FGF4 and heparin (Figures 1A,B and S1A). Among these TSC lines, the majority of colonies derived from 10 lines displayed a flattened morphology during long‐term culture. Unexpectedly, two cell lines exhibited typical compact, dome‐shaped colony morphology and could be stably passaged for over 40 passages in conventional TSC medium (Figures 1C,D and S1A). Given that the colony morphology of these two TSC lines resembled that of mouse naïve ESCs [28], we cultured mouse ESCs in conventional TSC medium. These ESCs could not be maintained under this condition beyond 6 days (Figure S1B), which is highly consistent with previous studies [44, 45]. Together, these findings suggested that we isolated a novel subtype of dome‐shaped TSCs, termed dTSCs, with a morphology distinct from that of cTSCs.

To further estimate the characteristics of dTSCs, RNA‐seq was performed. Global transcriptional clustering analysis revealed that dTSCs were similar to cTSCs but distinct from ESCs (Figure 1E and Data S1). GO enrichment analysis also verified the activation of genes related to ‘embryonic placenta development’ in both dTSCs and cTSCs (Figure 1F and Data S2). Marker genes of TSCs (*Cdx2*, *Gata3*, *Tfap2c*, *Eomes*, *Elf5*, and *Tead4*) displayed significant enrichment in dTSCs, whereas they barely expressed the marker genes of ESCs (*Oct4* and *Nanog*) (Figure 1G,H and Data S1). Moreover, though dTSCs propagated slower than cTSCs, the proliferation capacity (Ki67), cell cycle, and karyotype of dTSCs showed no obvious difference from those of cTSCs (Figures S1C–S1H). In sum, these results indicated that dTSCs possessed the representative molecular features of TSCs.

### 3.2 dTSCs Exhibit Relatively Low Cellular Heterogeneity and Limited Spontaneous Differentiation Under Stem Cell Culture Conditions

**FIGURE 2:**
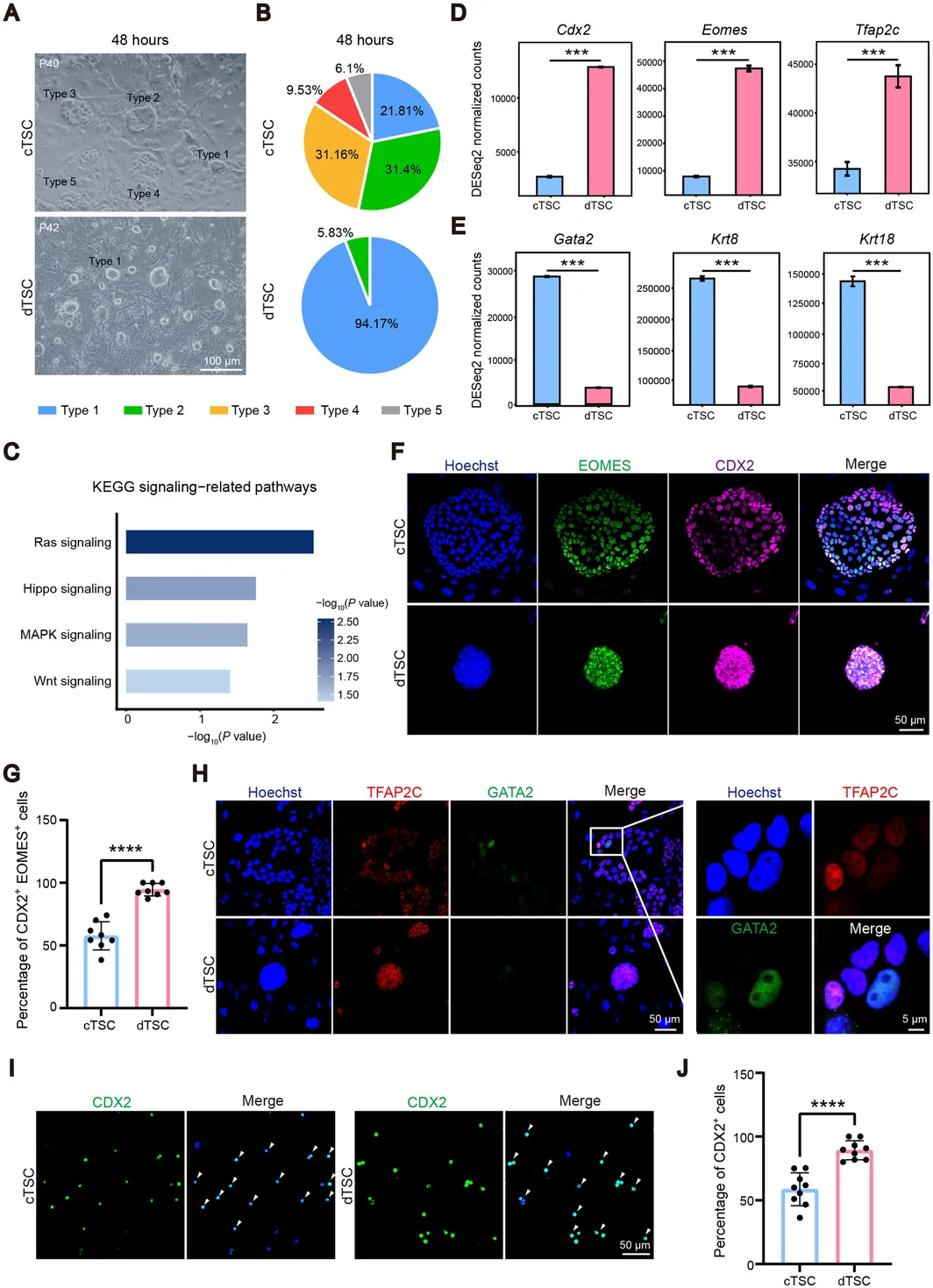
dTSCs exhibit relatively low cellular heterogeneity and limited spontaneous differentiation potential under stem cell culture conditions. (A) Representative colony morphology of P40 cTSCs and P42 dTSCs 48 h post‐passaging. Scale bar, 100 μm. (B) Pie charts showing the percentages of five types of TSC colonies corresponding to (A). (C) KEGG pathway enrichment analysis of up‐regulated DEGs in dTSCs relative to cTSCs. DEGs were identified with log_2_(fold change) > 1 and *p* < 0.05. (D–E) Bar plots showing the expression levels of pTE (D) and mTE (E) related genes, normalized to DESeq2‐normalized read counts. ***p < 0.001. (F) Immunofluorescence staining of CDX2 (pink) and EOMES (green) in cTSCs and dTSCs. The nuclei are stained with Hoechst 33342 (blue). Scale bar, 50 μm. (G) The percentage of CDX2 and EOMES double‐positive cells in cTSCs and dTSCs corresponding to (E). Data are presented as mean ± SD (*n* = 8); unpaired two‐tailed Student's *t*‐test; ****p < 0.0001. (H) Immunofluorescence staining of TFAP2C (red) and GATA2 (green) in cTSCs and dTSCs. The nuclei are stained with Hoechst 33342 (blue). Scale bars are shown as indicated. (I) Immunofluorescence staining of CDX2 (green) in single cells of cTSCs and dTSCs. The nuclei are stained with Hoechst 33342 (blue). Scale bar, 50 μm. (J) The percentage of cells with CDX2‐positive cells in cTSCs and dTSCs corresponding to (H). Data are presented as mean ± SD (*n* = 9); unpaired two‐tailed Student's *t*‐test; ****p < 0.0001.

Considering the notoriously heterogeneous nature of cTSCs [20], we wondered whether dTSCs have higher homogeneity. First, colonies of both cTSCs and dTSCs were classified into four major types as previously described: Type 1 was compact and dome‐shaped, Type 4 was flattened with a large multilayered cell cluster, and Types 2 and 3 were their intermediates. Analysis of colony morphology proportions showed that the colony morphology of cTSCs in our study was highly heterogeneous, which is consistent with previous studies [20]. Excitingly, the percentage of Type 1 colonies in dTSCs reached nearly 95% on feeder layers, indicating a high degree of morphological homogeneity (Figure 2A,B).

Previously, Motomura et al. had established that TSCs presented in Type 1 colonies can maintain their stem‐cell state indefinitely [20]. Consistently, DEG analysis between dTSCs and cTSCs revealed that several classical signalling pathways, such as Ras, MAPK, and Hippo signalling pathways, were specifically activated in dTSCs (Figures 2C and S2A; Data S3 and S4). It had been revealed that these pathways are essential for the maintenance of stem cell pluripotency, trophectoderm lineage differentiation, and placental development [46, 47, 48, 49, 50]. In addition, genes involved in regulating trophoblast self‐renewal (*Cdx2*, *Eomes*, *Tfap2c*, *Elf5*, and *Tead4*) and pTE markers (*Ly6a*, *Ddah1*, *Ets2*, *Gsto1*, and *Pard3*) were upregulated in dTSCs compared with trophectoderm (Figures 2D and S2B). Meanwhile, dTSCs exhibited significantly lower expression levels of differentiation and mural trophectoderm (mTE) markers (*Gata2*, *Krt8*, *Krt18*, *Klf9*, *Tfap2a*, *Rhox6*, *Rhox9*, *Ascl2*, *Slc2a3*, and *Dppa1*) (Figures 2E and S2C). In situ immunostaining further confirmed a higher proportion of CDX2 and EOMES double‐positive (CDX2^+^EOMES^+^) and TFAP2C‐positive cells, along with almost undetectable GATA2 signals in dTSCs (Figure 2F–H). Moreover, when TSC colonies were dissociated into single cells, we observed that the percentage of CDX2‐positive cells in dTSCs (nearly 90%) was substantially higher than that in cTSCs (nearly 60%) (Figure 2I,J).

Previously, trophectoderm stem cells (TESCs) [23] and morula‐derived trophoblast ectodermal stem cells (MTSCs) [51] were identified as two types of TSCs with a highly self‐renewal state or enhanced trophoblast core gene expression. In addition, we found that cell growth, cell adhesion, as well as MAPK and PI3K signalling pathways were activated in dTSCs, TESCs, and MTSCs. Conversely, these cells exhibited lower differentiation potential than cTSCs, as indicated by common down‐regulation of genes involved in epithelial‐mesenchymal transition (EMT), cell fate commitment, and nervous system processes (Figures S3A–S3D; [Link], [Link]). Collectively, these findings suggested that dTSCs, like other high‐quality TSCs (TESCs and MTSCs), exhibited relatively low cellular heterogeneity and limited spontaneous differentiation potential under stem cell conditions compared with cTSCs.

### 3.3 dTSCs Have an Enhanced Adhesion Capacity

**FIGURE 3:**
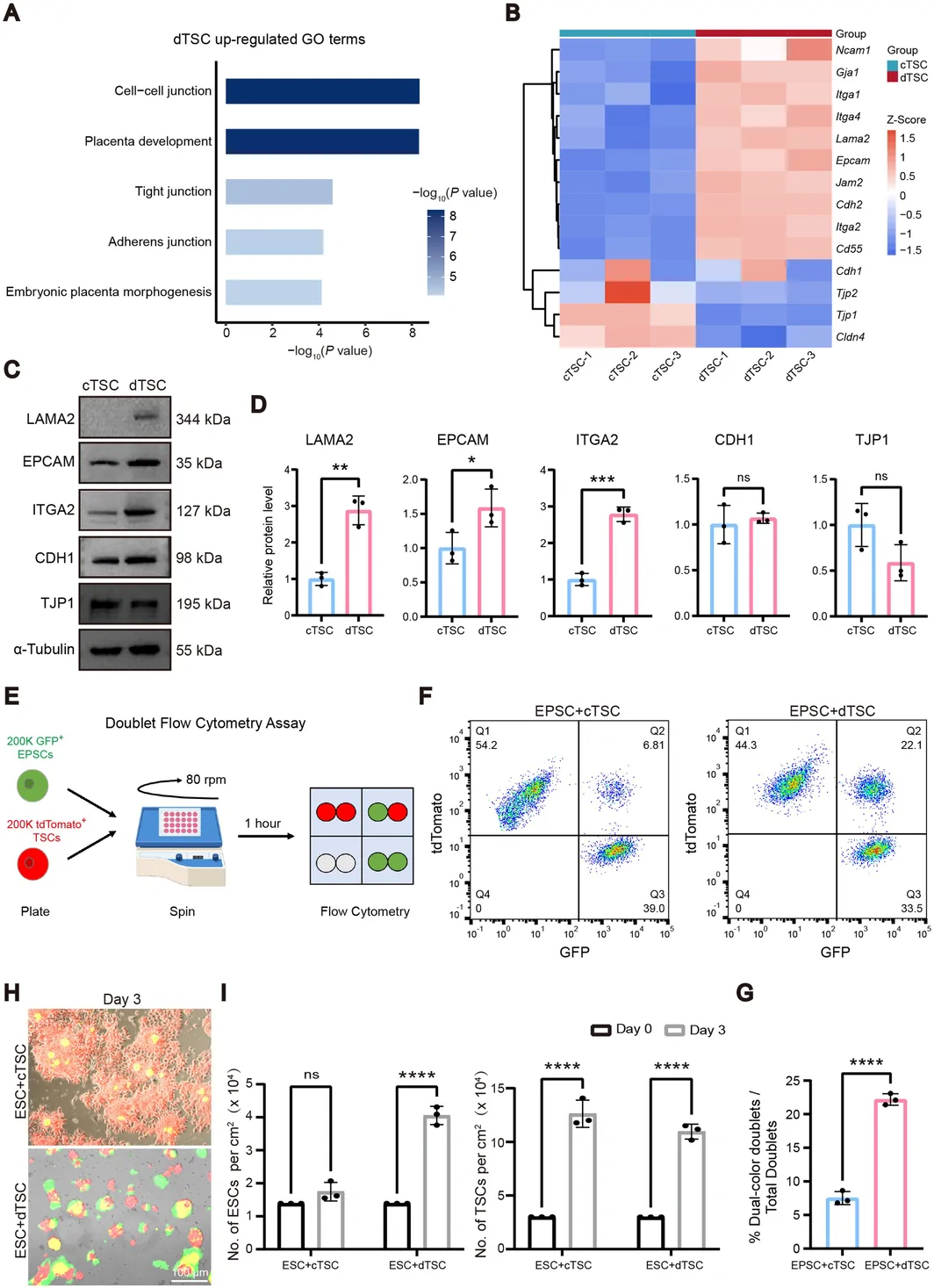
dTSCs have an enhanced adhesion capacity. (A) GO enrichment analysis of up‐regulated DEGs in dTSCs relative to cTSCs. The colour gradient indicates the significance level (*p*‐value). The criteria for DEGs are defined as log_2_(fold change) > 1 and *p* < 0.05. (B) Heatmap analysis of cell junction‐related DEGs between cTSCs and dTSCs. The colour key from blue to red indicates low to high gene expression levels, respectively. Data are *Z*‐score transformed prior to clustering. (C) Western blotting analysis of LAMA2, EPCAM, ITGA2, CDH1, and TJP1 in cTSCs and dTSCs, with α‐Tubulin as the internal control. (D) The relative expression levels of the proteins in (C). Data are presented as mean ± SD (*n* = 3 biological replicates); unpaired two‐tailed Student's *t*‐test; *p < 0.05, **p < 0.01, ***p < 0.001. (E) Schematic of the doublet flow cytometry assay. (F) Representative results of the doublet flow cytometry assay for mouse GFP‐EPSCs + tdTomato‐cTSCs and GFP‐EPSCs + tdTomato‐dTSCs. (G) Quantification of cell adhesion via doublet flow cytometry. The percentage of dual‐coloured doublets indicates the adhesion between GFP‐EPSCs and tdTomato‐labelled dTSCs or cTSCs. Data are presented as mean ± SD (*n* = 3 biological replicates); unpaired two‐tailed Student's *t*‐test; ****p < 0.0001. (H) Representative fluorescence and bright field merged images illustrate the progression of FTW‐ESCs (green) in co‐culture with FTW‐TSCs (red) on Day 3. Scale bar, 50 μm. (I) Cell numbers from Day 3 for FTW‐ESCs co‐cultures with FTW‐cTSCs or dTSCs. Data are presented as mean ± SD (*n* = 3 biological replicates); unpaired two‐tailed Student's *t*‐test; ****p < 0.0001.

Next, we further explored the unique transcriptional signature of dTSCs. By performing GO term analysis on the DEGs between dTSCs and cTSCs, we found that genes related to cell–cell junctions were significantly enriched in dTSCs (Figure 3A, Data S3 and S5). Specifically, molecules associated with cell adhesion (e.g., *Ncam1*, *Cdh2*, *Gja1*, *Epcam*, and *Jam2*) and extracellular matrix (e.g., *Lama2*, *Itga1*, *Itga2*, and *Itga4*) were highly expressed in dTSCs, consistent with the typically higher cell density observed in dTSC colonies (Figure 3B,D and Data S3).

Recently, TSCs have emerged as an ideal model for investigating crosstalk between stem cells of embryonic and extraembryonic origins [29, 52, 53]. To assess whether enhanced cell adhesion in dTSCs promotes robust cell–cell junctions between TSCs and pluripotent stem cells, we adapted the doublet flow cytometry assay from a previous report [37]. This assay quantifies the proportion of cell doublets comprising one red and one green cell derived from the same or distinct cell types (Figure 3E). Compared with tdTomato‐cTSCs, tdTomato‐dTSCs exhibited significantly enhanced adhesion to mouse GFP‐EPSCs (Figure 3F,G). More interestingly, when ESCs and TSCs were co‐cultured in FTW medium [38], although both dTSCs and cTSCs proliferated at comparable rates, dTSCs interacted more closely with ESCs and better supported their proliferation (Figure 3H,I), implying enhanced communication between ESCs and dTSCs relative to that between ESCs and cTSCs.

### 3.4 dTSCs Can Differentiate Into Mature Trophoblast Cells In Vitro

**FIGURE 4:**
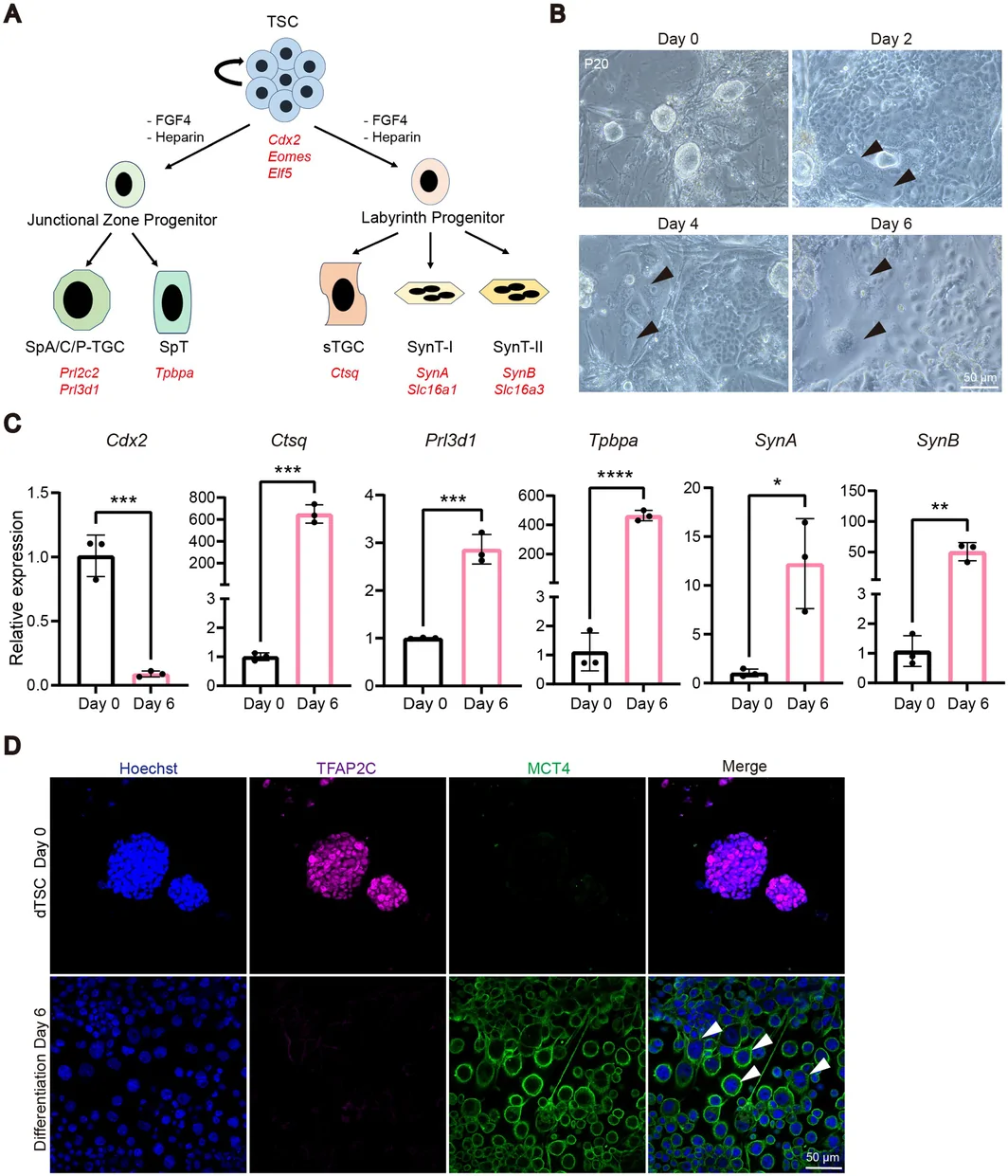
dTSCs can differentiate into mature trophoblast cells in vitro. (A) Simplified diagram of the main differentiation trajectories of mouse trophoblast stem cells (TSCs) and pertinent markers of differentiated cell types. SpT, spongiotrophoblast; SpA‐TGC, spiral artery‐associated trophoblast giant cell; c‐TSC, canal trophoblast giant cell; P‐TGC, parietal trophoblast giant cell; sTGC, sinusoidal trophoblast giant cell; SynT‐I, syncytiotrophoblast layer I cell; SynT‐II, syncytiotrophoblast layer II cell. (B) Phase‐contrast images of P20 dTSCs differentiated for 2, 4, and 6 days in differentiation medium, respectively. Representative nuclei of differentiated giant cells are indicated by the black arrowheads. Scale bar, 50 μm. (C) Relative mRNA expression of *Cdx2*, *Ctsq*, *Prl3d1*, *Tpbpa*, *SynA*, and *SynB* in differentiating dTSCs at Day 6. qPCR data are normalized to *Gapdh* and presented as mean ± SD (*n* = 3 biological replicates); unpaired two‐tailed Student's *t*‐test; *p < 0.05, **p < 0.01, ***p < 0.001, and ****p < 0.0001. (D) Immunofluorescence staining of TFAP2C (pink) and MCT4 (green) in dTSCs after 6 days of differentiation. Cell nuclei are stained with Hoechst 33342 (blue). Scale bar, 50 μm.

In the absence of FGF4, TSCs randomly differentiate into various trophoblast cell subtypes in vitro, including trophoblast giant cells (TGCs), parietal TGCs (P‐TGCs), sinusoidal TGCs (S‐TGCs), spiral associated TGCs (SpA‐TGCs), canal TGCs (C‐TGCs), spongiotrophoblasts (SpTs), syncytiotrophoblast layer I (SynT‐I), and syncytiotrophoblast layer II (SynT‐II) (Figure 4A) [54, 55, 56, 57]. When dTSCs were cultured in TSC medium without FGF4 and heparin, large TGCs emerged as early as Day 2 (Figure 4B). On Day 6 of in vitro differentiation, the expression of TSC marker genes *Cdx2* and *Tfap2c* significantly decreased, accompanied by increased expression of marker genes of TGCs (*Ctsq* and *Prl3d1*), SpT (*Tpbpa*), SynT (*SynA* and *SynB*), and mature trophoblast cells (MCT4) (Figure 4C,D). These time courses of in vitro differentiation were highly consistent with those observed in cTSCs (Figure S4A,B).

### 3.5 dTSCs Can Differentiate Into Placental Terminal Trophoblast Cells In Vivo

**FIGURE 5:**
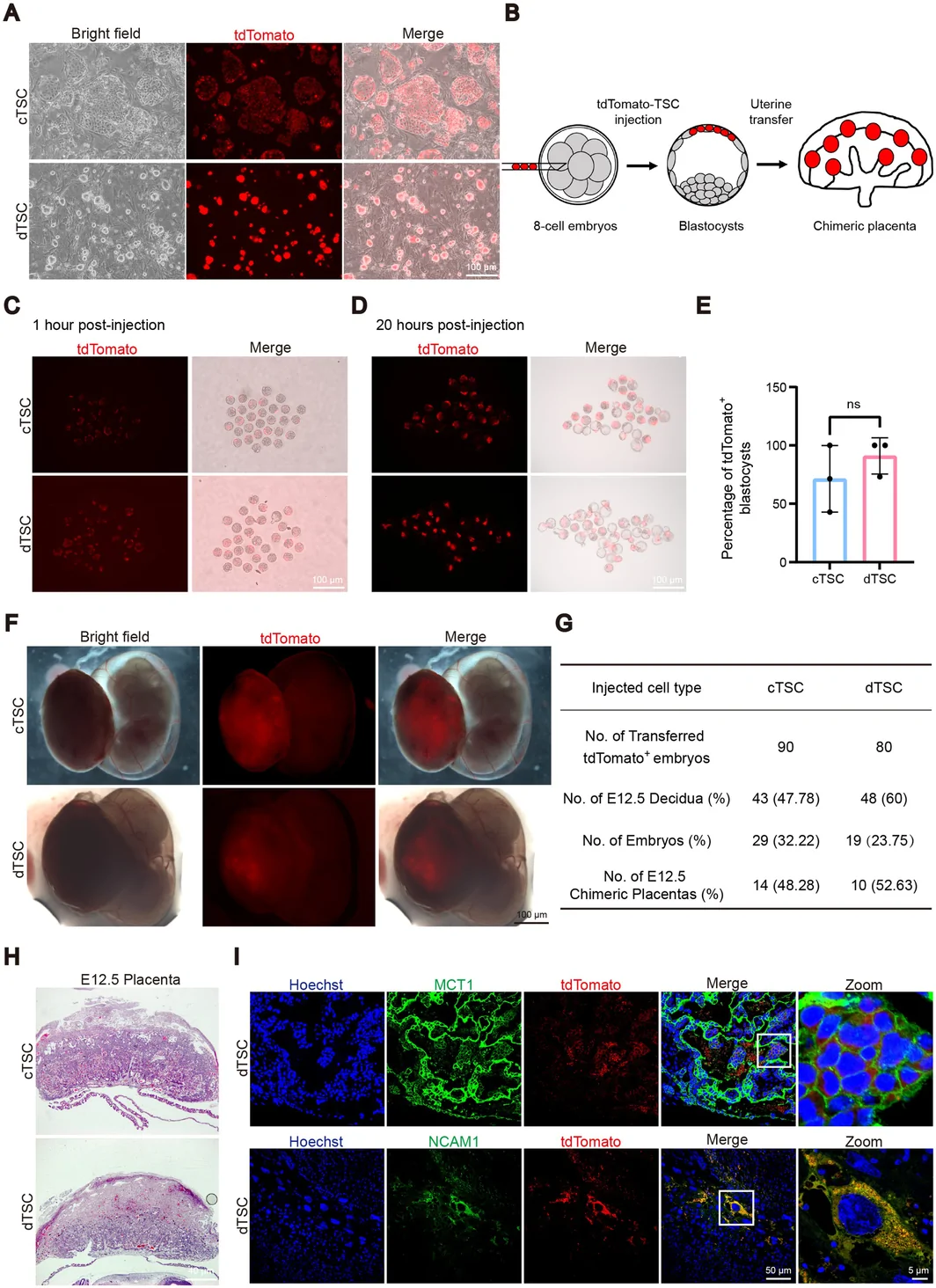
dTSCs can differentiate into placental terminal trophoblast cells in vivo. (A) Representative images of P14 cTSCs and P12 dTSCs carrying tdTomato fluorescence. Scale bar, 100 μm. (B) Schematic diagram illustrating the generation of chimeric placentas by injecting tdTomato‐labelled TSCs into 8‐cell stage embryos. (C) Representative images of chimeric morula‐stage embryos generated by injecting 10 tdTomato‐labelled cTSCs or dTSCs into 8‐cell stage embryos. Scale bar, 100 μm. (D) Representative images of chimeric blastocysts generated by injecting 10 tdTomato‐labelled cTSCs or dTSCs into 8‐cell stage embryos. Scale bar, 100 μm. (E) Statistical analysis of the proportion of tdTomato‐positive blastocysts. Data are presented as mean ± SD (*n* = 3 biological replicates); unpaired two‐tailed Student's *t*‐test; ns, not significant. (F) Contribution of tdTomato‐labelled cTSCs and dTSCs to placenta formation in E12.5 conceptuses. (G) Data listing detailed information on chimeric‐embryo formation. (H) Histological analysis of E12.5 chimeric mouse placentas by H&E staining. Scale bar, 40 μm. (I) Immunofluorescence staining of MCT1 (green, top) and NCAM1 (green, bottom) in E12.5 chimeric placentas (tdTomato‐labelled). Cell nuclei are stained with Hoechst 33342 (blue). Scale bars are shown as indicated.

Furthermore, tdTomato‐dTSCs were injected into 8‐cell stage embryos, followed by in vitro blastocyst development and subsequent in vivo transplantation into pseudopregnant mice to assess their differential potential (Figure 5A–D). The results showed that nearly 80% of morulae injected with dTSCs or cTSCs could develop into blastocysts in vitro, indicating that dTSC injection did not hamper embryonic development before transplantation. Meanwhile, no significant difference was found in the proportions of tdTomato‐positive blastocysts and E12.5 placentas following dTSC or cTSC injection, indicating that dTSCs possessed intact developmental integration capacity during early embryonic development (Figure 5E–G). To further identify the trophoblast lineages that dTSCs contribute to in the placenta, H&E staining was first performed to characterise the histological structure of E12.5 placentas, followed by immunofluorescence staining for the junctional zone marker NCAM1 [24, 58] and syncytiotrophoblast marker MCT1 [59, 60], together with tdTomato‐labelled dTSCs (Figure 5H–I). These findings demonstrated that dTSCs were capable of differentiating into mature placental trophoblast lineages in vivo.

### 3.6 *Foxo4* and *Slc16a3* Are Essential for dTSCs‐Mediated Placental Development

**FIGURE 6:**
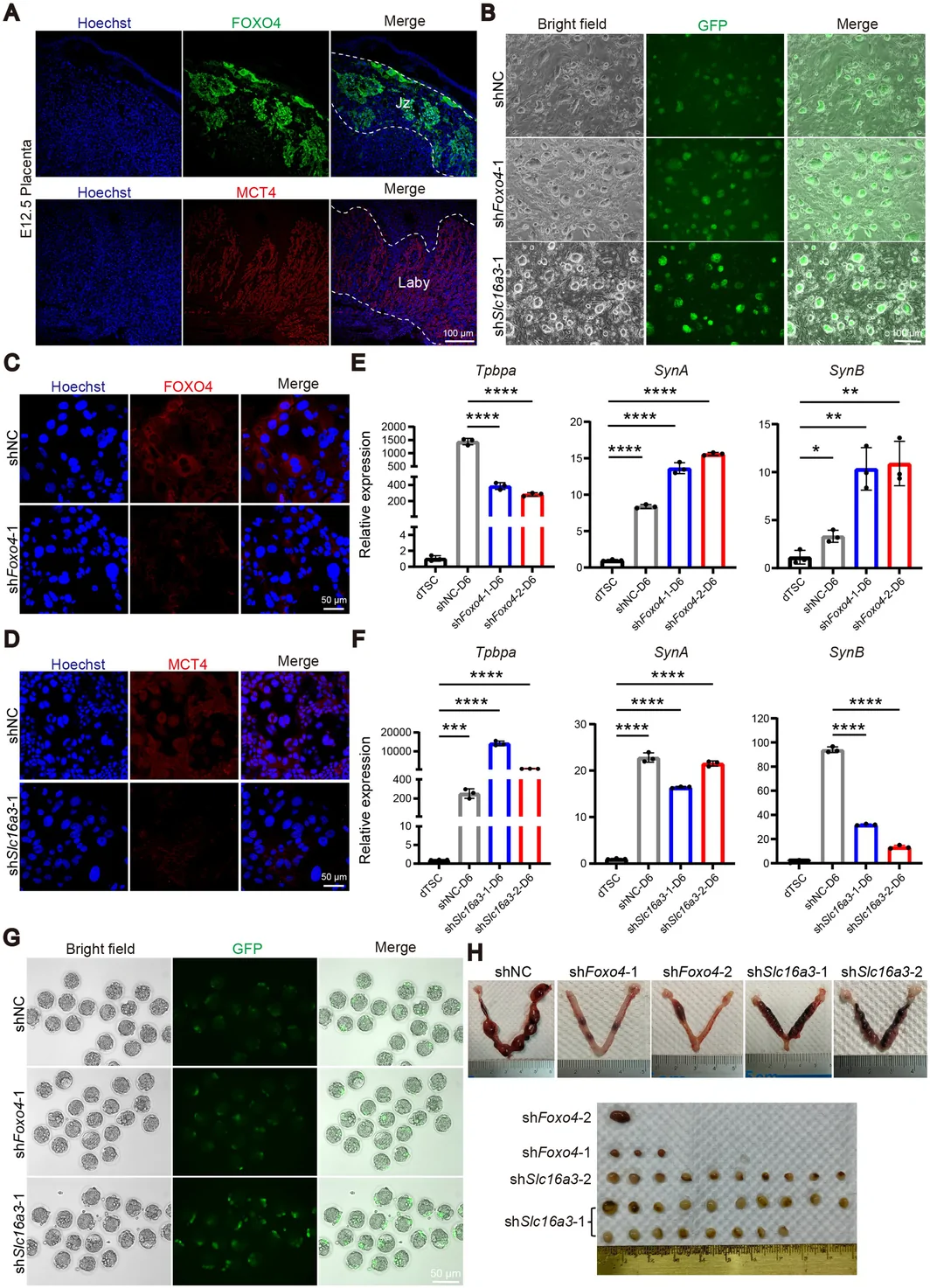
*Foxo4* and *Slc16a3* are essential for dTSCs‐mediated placental development. (A) Immunofluorescence staining of FOXO4 (green) and MCT4 (red) in the E12.5 placenta. Cell nuclei are stained with Hoechst 33342 (blue). Jz, Junctional zone; Laby, Labyrinth. Scale bar, 100 μm. (B) Cell morphology of knockdown *Foxo4* and *Slc16a3* dTSCs (GFP‐labelled). Scale bar, 100 μm. (C) Immunofluorescence staining of FOXO4 (red) in dTSCs and *Foxo4*‐KD dTSCs at Day 6 of differentiation. Cell nuclei are stained with Hoechst 33342 (blue). Scale bar, 50 μm. (D) Immunofluorescence staining of MCT4 (red) in dTSCs and *Slc16a3*‐KD dTSCs at Day 6 of differentiation. Cell nuclei are stained with Hoechst 33342 (blue). Scale bar, 50 μm. (E) Relative mRNA expression of *Tpbpa*, *SynA*, and *SynB* in *Foxo4*‐KD dTSCs versus negative controls during differentiation. Data are presented as mean ± SD (*n* = 3 biological replicates); one‐way ANOVA; *p < 0.05, **p < 0.01, ****p < 0.0001. (F) Relative mRNA expression of *Tpbpa*, *SynA*, and *SynB* in *Slc16a3*‐KD dTSCs versus negative controls during differentiation. Data are presented as mean ± SD (*n* = 3 biological replicates); one‐way ANOVA; ***p < 0.001, ****p < 0.0001. (G) Formation of chimeric blastocysts following the injection of 10 GFP‐labelled dTSCs (Control, *Foxo4*‐KD, or *Slc16a3*‐KD) into 8‐cell stage embryos. Scale bar, 50 μm. (H) E12.5 deciduae derived from chimeric embryos.

*Foxo4* and *Slc16a3* (encoding MCT4) were identified as marker genes specifically expressed in the junctional zone and labyrinthine layer within the E12.5 mouse placentas, respectively (Figure 6A) [59, 61, 62]. However, the in vivo developmental potential of mouse TSCs lacking these two genes has not been investigated. To explore the functions of *Foxo4* and *Slc16a3* during placental development, we generated *Foxo4* and *Slc16a3* knockdown (*Foxo4*‐KD and *Slc16a3*‐KD) dTSCs labelled with GFP using shRNAs (Figures 6B, S5A and S5B). When dTSCs differentiated in vitro, the protein levels of *Foxo4* and *Slc16a3* were dramatically decreased (Figure 6C,D and S5C,D). Meanwhile, the expression of the spongiotrophoblast marker *Tpbpa* was reduced in *Foxo4*‐KD cells compared with that in controls (Figure 6E). By contrast, *SynA* and *SynB*, two marker genes of labyrinthine trophoblasts, maintained their expression in *Foxo4*‐KD trophoblast cells (Figure 6E). Moreover, *SynB* was significantly decreased, while the expression of *Tpbpa* and *SynA* remained at a high level in *Slc16a3*‐KD trophoblast cells compared with controls (Figure 6F). Subsequently, *Foxo4*‐KD and *Slc16a3*‐KD dTSCs were injected into 8‐cell stage embryos to investigate their in vivo developmental potential. Although they were able to contribute to blastocysts, *Foxo4*‐KD and *Slc16a3*‐KD dTSCs failed to support the development of live foetuses or normal placentas (Figure 6G, Figures S5E and 6H). Together, these findings verified the critical role of *Foxo4* and *Slc16a3* in dTSCs, as well as their functions in regulating placental development.

## 4 Discussion and Conclusion

In this study, we successfully established a novel trophoblast stem cell line termed dTSCs derived from mouse preimplantation blastocysts. Compared with cTSCs, dTSCs exhibited higher stemness, a markedly lower differentiation tendency, and reduced heterogeneity.

Considering that the dTSCs were derived from E3.5 blastocysts using the method for establishing cTSCs, we would like to propose several explanations for why the dTSCs could be obtained in the present study: (1) Mouse embryo polarisation is asynchronous at the 8‐cell stage, and this heterogeneous timing of embryo polarisation may be linked to earlier heterogeneities in the embryo. In addition, early polarisation upregulates the expression of trophectoderm determinants (CDX2) [63]. Therefore, it is suggested that the heterogeneity between embryos may persist into the blastocyst stage. (2) Previously, single‐cell RNA sequencing and immunofluorescence staining of the mouse blastocysts demonstrated that the expression level of stemness‐related genes in polar trophectoderm trophoblast cells near the inner cell mass (ICM) was highly heterogenous [23, 64, 65]. These findings were consistent with the observations through three‐dimensional real‐time imaging of early embryos, which revealed a spatiotemporal gradient in ERK activity in trophoblast cells, where ERK activity regulates *Cdx2* expression [66]. Together, these findings suggest the possibility that dTSCs and cTSCs may be derived from embryos at different statuses, respectively.

Recently, Rivron NC and Zhang labs reported that TESCs [23] and MTSCs [51] both exhibited a more homogeneous expression of *Cdx2* and significantly higher expression of pTE‐related genes (e.g., *Tead4*, *Elf5*, *Eomes*, and *Ddah1*) compared with cTSCs. Of note, the protein level of *Tfap2c* was unchanged though its mRNA was increased in dTSCs (Figures 1H and 2C). This discrepancy may reflect the activated regulation of translational and post‐translational mechanisms in dTSCs. Furthermore, dTSCs also expressed a higher level of these representative markers of pTE than cTSCs (Figures 2C and S2A). Considering the heterogeneity of different embryos at the same stage or the trophoblasts within an embryo, this implies that dTSCs may be derived from the trophoblast at a more primitive state.

Although different types of colonies have been identified in cTSCs previously, there was no global dissection of the expression differences between Type 1 and other types of colonies [20]. In our study, dTSCs were established, with the proportion of Type 1 TSCs colonies reaching 95%. RNA‐seq analysis between dTSCs, TESCs, MTSCs, and cTSCs provided a comprehensive transcriptional characterisation (especially the key activated signalling pathways) of the Type 1 colonies of TSCs with an enhanced self‐renewal capacity, thus providing an important reference for establishing a new culture system to obtain high‐quality TSCs. Rivron NC et al. previously demonstrated that the level of trimethylation of H3K4 (H3K4me3) was much higher in the CDX2^high^ TSCs compared to that in the CDX2^low^ TSCs [23], suggesting that the epigenetic characteristics of dTSCs may be significantly different from those of cTSCs, contributing to their stronger self‐renewal ability. Thus, it is worthwhile to further investigate the epigenetic regulatory mechanisms underlying the enhanced self‐renewal of dTSCs to improve the culture medium and quality of TSCs.

The aggregation of TSCs with ESCs [67] or EPSCs [29] in vitro reconstructs blastocyst‐like structures, serving as an ideal model for studying early embryogenesis. Previous studies have demonstrated that cell adhesion directly influences the efficiency of pluripotent stem cells integrating into xenogeneic embryos [37, 68, 69]. In our study, after temporarily co‐culturing TSCs with EPSCs, we found that the aggregation capacity of dTSCs with EPSCs was higher than that of cTSCs. Additionally, through co‐culturing ESCs with TSCs, we also discovered that dTSCs could better support the self‐renewal of ESCs. Furthermore, although no significant difference was detected, we still observed that the ratio of dTSCs contributing to the blastocyst was slightly higher than that of cTSCs (Figure 5E). In conclusion, these results collectively suggest that dTSCs may have better communication with pluripotent stem cells and the ICM during embryo development.

In terms of the application of dTSCs, trophoblast organoids have recently emerged as a favourable model for investigating placental development in vitro [51, 70, 71]. Therefore, the aforementioned advantages and characteristics of dTSCs also provide a good source of stem cells for the future establishment of trophoblast organoids. Moreover, *Foxo4* (a spongiotrophoblast marker) and *Slc16a3* (a syncytiotrophoblast marker) were knocked down in dTSCs to investigate their roles in trophoblast lineage development. This provides a paradigm for conducting genome‐wide genetic manipulation with dTSCs, thus providing an ideal model to investigate placental development and its underlying regulatory mechanisms.

## Author Contributions

M.W. and X.‐Y.Z. conceived and supervised the project. M.L., S.R, X.X., H.Y. and Y.Z. performed the experiments. G.L. and J.Z. performed bioinformatic analyses. M.L., M.W. and X.‐Y.Z. wrote the manuscript. All authors contributed to the manuscript revision, and approved the submitted version.

## Funding

This work was supported by the National Key Research and Development Program of China (2022YFA1106200, 2022YFA0806303, 2024YFA1802602), the National Natural Science Foundation of China (U22A20278, 82530051, 32370911, 32571016), Guangzhou Key Research and Development Program (2024B03J0991), Guangdong‐Hong Kong Joint Laboratory for Psychiatric Disorders (2023B1212120004) and Guangdong Major Project of Basic Research (2025B0303000014).

## Conflicts of Interest

The authors declare no conflicts of interest.

## Supporting information


**Data S1:** Shared up‐regulated differentially expressed genes (DEGs) in dTSCs and cTSCs compared with ESCs.

**Data S2:** GO term enrichment analysis of up‐regulated DEGs in TSCs versus ESCs based on RNA‐seq data.

**Data S3:** Up‐regulated DEGs in dTSCs versus cTSCs.

**Data S4:** KEGG analysis of up‐regulated DEGs in dTSCs versus cTSCs based on RNA‐seq data.

**Data S5:** GO term enrichment analysis of up‐regulated DEGs in dTSCs versus cTSCs based on RNA‐seq data.

**Data S6:** DEGs from dTSC vs. cTSC and TESC vs. cTSC comparisons.

**Data S7:** Shared up‐regulated DEGs from dTSC vs. cTSC and TESC vs. cTSC comparisons.

**Data S8:** Shared down‐regulated DEGs from dTSC vs. cTSC and TESC vs. cTSC comparisons.

**Data S9:** GO enrichment for shared up‐regulated DEGs from dTSC vs. cTSC and TESC vs. cTSC comparisons based on RNA‐seq data.

**Data S10:** GO enrichment for shared down‐regulated DEGs from dTSC vs. cTSC and TESC vs. cTSC comparisons based on RNA‐seq data.

**Data S11:** DEGs from dTSC vs. cTSC and MTSC vs. cTSC comparisons.

**Data S12:** Shared up‐regulated DEGs from dTSC vs. cTSC and MTSC vs. cTSC comparisons.

**Data S13:** Shared down‐regulated DEGs from dTSC vs. cTSC and MTSC vs. cTSC comparisons.

**Data S14:** GO enrichment for shared up‐regulated DEGs from dTSC vs. cTSC and MTSC vs. cTSC comparisons based on RNA‐seq data.

**Data S15:** GO enrichment for shared down‐regulated DEGs from dTSC vs. cTSC and MTSC vs. cTSC comparisons based on RNA‐seq data.

**Table S1:** The information of antibodies used in this study.
**Table S2:** The sequences of qPCR primers used in this study.
**Table S3:** shRNA sequences used in this study.
**Figure S1:** Cell line summary and characterisation of proliferation, cell cycle and karyotype in dTSCs and cTSCs. Related to Figure 1.
**Figure S2:** Heatmap of signalling‑pathway DEGs and comparison of pTE/mTE marker gene expression between dTSCs and cTSCs. Related to Figure 2.
**Figure S3:** Venn Plots and GO analysis of DEGs from dTSCs, TESCs and MTSCs versus cTSCs. Related to Figure 2.
**Figure S4:** cTSCs can differentiate into mature trophoblast cells in vitro. Related to Figure 4.
**Figure S5:** Characterisation of *Foxo4*‑KD and *Slc16a3*‑KD dTSCs and generation of chimeric blastocysts. Related to Figure 6.

## Data Availability

The accession number for the RNA‐seq data reported in this paper is Gene Expression Omnibus (GEO): GSE315936. Raw data of GSE175644, GSE245037, GSE90752 and PRJNA1092867 are cited in this article.
