# Supplementary material for: Derivation of Mouse Dome‐Shaped Trophoblast Stem Cells With Low Heterogeneity From Blastocysts
Source: Cell Prolif. 2026 Sep 18:e70280. Online ahead of print. doi: 10.1111/cpr.70280 (PMC13586697; doi:10.1111/cpr.70280)
Supplement: Supplementary file 1 [file CPR-9999-e70280-s013.zip › cpr70280-sup-0016-Supinfo16.docx]

**Supporting Information**

**Derivation of Mouse Dome-Shaped Trophoblast Stem Cells with Low Heterogeneity from Blastocysts**

Minli Liu^1, 9^, Shaofang Ren^1, 9^, Xinyu Xia^1, 9^, Guanlin Lu^1^, Haojie Yang^1^, Yi Zheng^1^, Jiexiang Zhao^8^, Xiao-Yang Zhao^1, 2, 3, 4, 5, 6, 7, *^, Mei Wang^1, *^

^1^State Key Laboratory of Organ Failure Research, Department of Developmental Biology, School of Basic Medical Sciences, Southern Medical University, Guangzhou, Guangdong, 510515, China.

^2^School of Laboratory Medicine and Biotechnology, Southern Medical University, Guangzhou, Guangdong, 510515, China.

^3^Key Laboratory of Mental Health of the Ministry of Education, Guangzhou, Guangdong, 510515, China.

^4^Guangdong Provincial Key Laboratory of Construction and Detection in Tissue Engineering, Southern Medical University, Guangzhou, Guangdong, 510515, China.

^5^Guangdong-Hong Kong Joint Laboratory for Psychiatric Disorders, Guangzhou, Guangdong, 510515, China.

^6^Department of Gynecology, Zhujiang Hospital, Southern Medical University, Guangzhou, Guangdong, 510280, China.

^7^National Clinical Research Center for Kidney Disease, Guangzhou, Guangdong, 510515, China.

^8^Key Laboratory of Tropical Translational Medicine of Ministry of Education, School of Basic Medical Sciences, Hainan Academy of Medical Sciences, Hainan Medical University, Haikou, Hainan, 571101, China.

^9^These authors contributed equally to this work.

^*^Correspondence: [zhaoxiaoyang@smu.edu.cn (X.Z.) /](mailto:zhaoxiaoyang@smu.edu.cn%20(X.Z.)%20/) [wangmei94@smu.edu.cn](mailto:wangmei94@smu.edu.cn) (M.W.)

**Table S1.** The information of antibodies used in this study.

| **Antibodies** | **Sources** | **Information** | **Application** |
| --- | --- | --- | --- |
| Ki67 | Abcam | Cat# ab15580; RRID: AB_443209 | IF: 1:500 |
| CDX2 | BioGenex | Cat# MU392A-UC; RRID: AB_3101998 | IF: 1:200 |
| EOMES | Abcam | Cat# ab216870; RRID: AB_2943124 | IF: 1:300 |
| TFAP2C | Santa Cruz | Cat# sc-377458 | IF: 1:50; WB: 1:500 |
| MCT4 | Proteintech | Cat# 22787-1-AP; RRID: AB_11182479 | IF: 1:200 |
| MCT1 | Proteintech | Cat# 20139-1-AP; RRID: AB_2878645 | IF: 1:200 |
| NCAM1 | Abcam | Cat# ab237708; RRID: AB_3676336 | IF: 1:50 |
| RFP | Rockland | Cat# 600-401-379; RRID: AB_2209751 | IF: 1:200 |
| FOXO4 | ZEN BIO | Cat# 380928; RRID: | IF: 1:100 |
| Alexa Fluor 488-conjugated goat anti-rabbit IgG | Jackson ImmunoResearch | Cat# 111-545-003; RRID: AB_2338046 | IF: 1:500 |
| Alexa Fluor 594-conjugated goat anti-rabbit IgG | Jackson ImmunoResearch | Cat# 111-585-003; RRID: AB_2338059 | IF: 1:500 |
| Alexa Fluor 488-conjugated goat anti-mouse IgG | Jackson ImmunoResearch | Cat# 115-545-003; RRID: AB_2338840 | IF: 1:500 |
| Alexa Fluor 594-conjugated goat anti-mouse IgG | Jackson ImmunoResearch | Cat# 115-585-003; RRID: AB_2338871 | IF: 1:500 |
| Alexa Fluor 647-conjugated goat anti-mouse IgG | Jackson ImmunoResearch | Cat# 115-605-003; RRID: AB_2338902 | IF: 1:500 |
| CDX2 | HUABIO | Cat# ET1605-4; RRID: AB_3069712 | WB: 1:2000 |
| GATA3 | Abcam | Cat# ab199428; RRID: AB_2819013 | WB: 1:1000 |
| OCT4 | Santa Cruz | Cat# sc-5279; RRID: AB_628051 | WB: 1:500 |
| TJP1/ZO-1 | Proteintech | Cat# 21773-1-AP; RRID: AB_10733242 | WB: 1:5000 |
| CDH1 | HUABIO | Cat# HA723564 | WB: 1:5000 |
| ITGA2 | Bioss | Cat# bsm-52613R | WB: 1:2000 |
| EPCAM | Bioss | Cat# bsm-52417R | WB: 1:2000 |
| LAMA2 | HUABIO | Cat# HA721532; RRID: AB_3072648 | WB: 1:1000 |
| α-Tubulin | ABclonal | Cat# AC014; RRID: AB_2772756 | WB: 1:2000 |
| HRP-conjugated goat anti-rabbit IgG | Proteintech | Cat# SA00001-2; RRID: AB_2722564 | WB: 1:5000 |
| HRP-conjugated goat anti-mouse IgG | Proteintech | Cat# SA00001-1; RRID: AB_2722565 | WB: 1:5000 |

**Table S2.** The sequences of qPCR primers used in this study.

| **Gene (Mouse)** | **Sequence (5’-3’)** |
| --- | --- |
| ms-*Cdx2*-F | AGTGAGCTGGCTGCCACACT |
| ms-*Cdx2*-R | GCTGCTGCTGCTTCTTCTTGA |
| ms-*Ctsq*-F | AATTGGCTATGGTTATGTGGGA |
| ms-*Ctsq*-R | TCACACAGTAGGGTATTGGG |
| ms-*Tpbpa*-F | ACTGGAGTGCCCAGCACAGC |
| ms-*Tpbpa*-R | GCAGTTCAGCATCCAACTGCG |
| ms-*Prl3d1*-F | CCCCTGTGTCATACTGCTTCC |
| ms-*Prl3d1*-R | TCCTGGGAGAGCCGTCAGT |
| ms-*SynA*-F | CCTCACCTCCCAGGCCCCTC |
| ms-*SynA*-R | GGCAGGGAGTTTGCCCACGA |
| ms-*SynB*-F | GGCAACTCTCCGCAGCTGACAC |
| ms-*SynB*-R | ACGAACCAGCTGTGCTTGAGCC |
| ms-*Gapdh*-F | TGTGAACGGATTTGGCCGTA |
| ms-*Gapdh*-R | ACTGTGCCGTTGAATTTGCC |

**Table S3.** shRNA sequences used in this study.

| **Plasmids** | **Sequences** |
| --- | --- |
| mouse *Foxo4* shRNA-1 | 5’-GACTTCAACTTTGAGCCAGAT-3’ |
| mouse *Foxo4* shRNA-2 | 5’-CAAGTTCATCAAGGTTCACAA-3’ |
| mouse *Slc16a3* shRNA-1 | 5’-CTGGGCTTCATCGACATCTTT-3’ |
| mouse *Slc16a3* shRNA-2 | 5’-GCTGGATGCAACCAAAGTTTA-3’ |
| mouse NC-shRNA | 5′- GGTTCTCCGAACGTGTCACGT-3′ |

**Figure S1. Cell line summary and characterization of proliferation, cell cycle and karyotype in dTSCs and cTSCs. Related to Figure 1.**

(A) Summary of the established cTSC and dTSC lines.

(B) Morphological changes of P14 ESCs during induction in TSC medium. Scale bar, 50 µm.

(C) Immunofluorescence staining of Ki67 (green) in cTSCs and dTSCs. Cell nuclei are stained with Hoechst 33342 (blue). Scale bar, 50 µm.

(D) Cell numbers of cTSCs and dTSCs cultured in TSC medium were quantified daily from day 0 (immediately after seeding) to 4. Data are shown as mean ± SD (n = 3 biological replicates); unpaired two-tailed Student’s *t* test; ^**^*P* < 0.01, ^****^*P* < 0.0001.

(E) Cell cycle analysis of P20 cTSCs and P18 dTSCs using propidium iodide staining and flow cytometric analysis.

(F) Quantitative analysis of cell cycle distribution in cTSCs and dTSCs. Data are presented as mean ± SD (n = 3 biological replicates); unpaired two-tailed Student’s *t* test; ns, not significant.

(G) Metaphase chromosome spreads of P15 cTSCs and P13 dTSCs. Images show the typical chromosomal complement of 40 chromosomes in both cell lines. Scale bar, 10 µm.

(H) Karyotypic chromosome counting of metaphase spreads in cTSCs and dTSCs. Data are presented as mean ± SD (n = 3 biological replicates); unpaired two-tailed Student’s *t* test; ns, not significant.

**Figure S2. Heatmap of signaling‑pathway DEGs and comparison of pTE/mTE marker gene expression between dTSCs and cTSCs. Related to Figure 2.**

(A) Heatmap analysis of signaling pathway-related DEGs between cTSCs and dTSCs. The color scale from blue to red indicates gradually increased gene expression. Data were Z-score transformed prior to clustering.

(B-C) Bar plots showing the expression level of pTE (B) and mTE (C) related genes, normalized to DESeq2-normalized read counts. ^**^*P* < 0.01, ^***^*P* < 0.001.

**Figure S3. Venn Plots and GO analysis of DEGs from dTSCs, TESCs and MTSCs versus cTSCs. Related to Figure 2.**

(A) Venn diagram analysis of DEGs obtained from dTSC versus cTSC and TESC versus cTSC comparisons.

(B) GO enrichment for shared up- and down-regulated DEGs from dTSC versus cTSC and TESC versus cTSC comparisons. The criteria for DEGs are defined as |log_2_(fold change)| > 1 and *P* < 0.05.

(C) Venn diagram analysis of DEGs obtained from dTSC versus cTSC and MTSC versus cTSC comparisons.

(D) GO enrichment for shared up- and down-regulated DEGs from dTSC versus cTSC and MTSC versus cTSC comparisons. The criteria for DEGs are defined as |log_2_(fold change)| > 1 and *P* < 0.05.

**Figure S4. cTSCs can differentiate into mature trophoblast cells *in vitro*. Related to Figure 4.**

(A) Relative mRNA expression of *Cdx2*, *Ctsq*, *Prl3d1*, *Tpbpa*, *SynA*, and *SynB* in differentiating cTSCs at Day 6. qPCR data are normalized to *Gapdh* and presented as mean ± SD (n = 3 biological replicates); unpaired two-tailed Student’s *t* test; ^*^*P* < 0.05, ^***^*P* < 0.001, and ^****^*P* < 0.0001.

(B) Immunofluorescence staining of TFAP2C (pink) and MCT4 (green) in cTSCs after 6 days of differentiation. Cell nuclei are stained with Hoechst 33342 (blue). Scale bar, 50 µm.

**Figure S5. Characterization of *Foxo4*‑KD and *Slc16a3*‑KD dTSCs and generation of chimeric blastocysts. Related to Figure 6.**

(A) Schematic illustration of shRNAs.

(B) Cell morphology of *Foxo4*-KD and *Slc16a3*-KD dTSCs (GFP-labeled). Scale bar, 100 µm.

(C) Immunofluorescence staining of FOXO4 (red) in dTSCs and *Foxo4*-KD dTSCs at Day 6 of differentiation. Cell nuclei are stained with Hoechst 33342 (blue). Scale bar, 50 µm.

(D) Immunofluorescence staining of MCT4 (red) in dTSCs and *Slc16a3*-KD dTSCs at Day 6 of differentiation. Cell nuclei are stained with Hoechst 33342 (blue). Scale bar, 50 µm.

(E) Formation of chimeric blastocysts following the injection of 10 GFP-labeled dTSCs (Control, *Foxo4*-KD, or *Slc16a3*-KD) into 8-cell stage embryos. Scale bar, 50 µm.
